# Molecular Modelling of the Adsorption and Delivery of α-Pinene and Similar Terpenes of Essential Oils on Montmorillonite Surfaces

**DOI:** 10.3390/nano15201573

**Published:** 2025-10-16

**Authors:** Shamsa Kanwal, Alfonso Hernández-Laguna, C. Ignacio Sainz-Díaz

**Affiliations:** 1 Department of Innovative Technologies in Medicine and Dentistry, University “G. d’Annunzio” of Chieti-Pescara, Via dei Vestini 31, 66100 Chieti, Italy; shamsa.kanwal@studenti.unich.it; 2 Instituto Andaluz de Ciencias de la Tierra, IACT-CSIC, Av. De las Palmeras, 4, 18100 Armilla, Spain; a.h.laguna@csic.es; 3 Departamento de Farmacia y Tecnología Farmacéutica, Facultad de Farmacia, Universidad de Granada, 18071 Granada, Spain

**Keywords:** molecular modelling, clay minerals, drug delivery, smectite, essential oils, DFT, force fields, molecular dynamics, IR spectroscopy, Raman spectroscopy

## Abstract

Alkylic molecules are found as some of the main components of natural essential oils. These essential oils offer several therapeutic properties in skin treatments and cosmetics. Systems providing controlled release of these molecules through the skin tissue are a challenge for their applications. This work explores some properties of the crystal structure of α-pinene and the adsorption and desorption of five terpenoid components of essential oils, such as α-pinene, limonene, β-ocimene, β-caryophyllene, and β-elemene, in the confined surfaces provided by natural clay minerals, particularly montmorillonite (MNT). These terpenoids have a methyl-ethenyl group as their common structural feature. Molecular modelling calculations have been applied at the atomic scale, including force fields, quantum mechanical methods, and molecular dynamics simulations. We calculated the crystallographic and spectroscopic properties of the α-pinene crystal via density functional theory (DFT)-level calculations, which were very close to the known experimental data. Moreover, this work explored the adsorption and desorption of these molecules in confined surfaces provided by MNT. Molecular dynamics simulations also showed the adsorption of these organics in the confined interlayer space of MNT at room temperature and allowed us to know the diffusion coefficient of these adsorbates in this material. The direct adsorption process of these molecules in the vapour phase is not energetically favourable, suggesting the use of non-aqueous solvents and kinetics and thermodynamic conditions for this process. However, the release of these molecules into aqueous media are energetically favourable, predicting that MNT–essential oil can be an excellent pharmaceutical formulation to be delivered in skin as a bioactive preparation with anti-inflammatory or cosmetic power. This research was performed to predict possible therapeutic applications for future experimental works.

## 1. Introduction

Essential oils are complex mixtures of volatile and aromatic compounds produced by plants, and they have been recognised for their therapeutic properties, particularly their anti-inflammatory, antimicrobial, and antioxidant activities [1]. Among the numerous constituents of essential oils, monoterpenes such as limonene, β-ocimene, β-elemene, and α-pinene, and the sesquiterpene β-caryophyllene, have attracted significant attention due to their potent anti-inflammatory effects. These molecules have a methyl-ethenyl group as their common structural feature.

**Figure 1:**
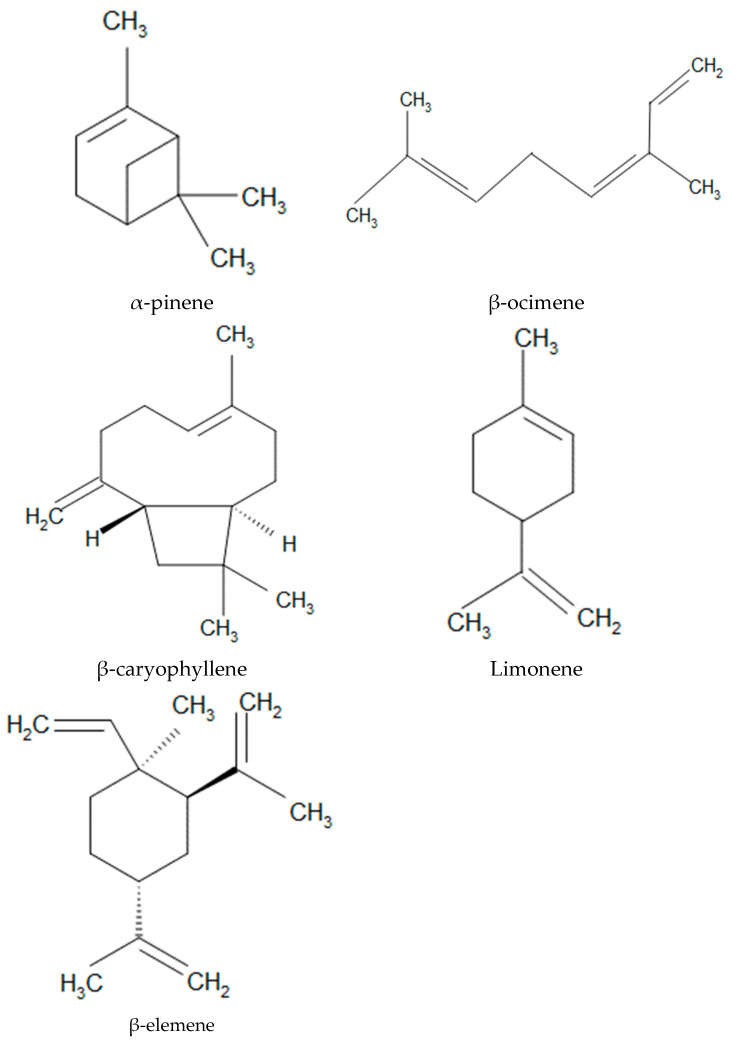
Molecular models of the studied terpenoid components of essential oils.

Limonene (Figure 1), a monocyclic monoterpene found abundantly in citrus oils, has demonstrated anti-inflammatory activity by downregulating pro-inflammatory cytokines such as TNF-α and IL-6 and inhibiting the activation of the NF-κB pathway [2]. The β-ocimene (Figure 1), another monoterpene commonly found in basil and lavender oils, has been used for inhibiting nitric oxide production and reducing inflammatory responses in vitro, although its mechanisms are less well defined compared with other terpenes [3]. α-pinene, a bicyclic monoterpene (Figure 1) widely distributed in pine and coniferous essential oils, exhibits both anti-inflammatory and bronchodilatory effects by suppressing COX-2 expression and prostaglandin E2 production [4]. β-caryophyllene, a bicyclic sesquiterpene (Figure 1) present in clove and cannabis oils, acts as a selective agonist of the cannabinoid receptor type 2 (CB2), thereby exerting immunomodulatory and anti-inflammatory effects without psychoactivity [5]. Similarly, β-elemene (Figure 1), a naturally occurring sesquiterpene commonly extracted from plants like Curcuma wenyujin, has demonstrated substantial anti-inflammatory and anticancer activities. β-elemene inhibits the production of inflammatory mediators such as TNF-α, IL-1β, and IL-6 by interfering with the NF-κB and MAPK signalling pathways [6]. β-elemene is a candidate of interest in various pharmaceutical formulations due to its multifaceted bioactivity, including those targeting chronic inflammation and tumour microenvironments [7].

Despite the pharmacological promise of these compounds, the clinical application of essential oils is limited by several physicochemical constraints. Notably, their high volatility, low aqueous solubility, and chemical instability under environmental and physiological conditions restrict their bioavailability and therapeutic efficacy [8]. Upon exposure to air or elevated temperatures, essential oil components readily evaporate, and many of them are susceptible to oxidation or degradation, making formulation into stable and controlled delivery systems a persistent challenge.

To address these limitations, various encapsulation and adsorption techniques have been explored to enhance the retention and bioavailability of volatile therapeutic compounds. Among these, the use of phyllosilicate clay minerals, particularly MNT, offers a promising strategy. Montmorillonite (MNT) is a 2:1 layered aluminosilicate with a high surface area, cation exchange capacity, and interlayer spacing that can accommodate a range of guest molecules [9]. Its structural features—comprising negatively charged tetrahedral–octahedral–tetrahedral (T-O-T) layers—make it well-suited for adsorption and intercalation of small organic compounds, thereby improving their thermal stability, volatility, resistance to oxidation, and controlled release profiles [10,11].

Recent studies have demonstrated the viability of clay–essential oil nanohybrids for this adsorption–desorption purpose [12,13]. For instance, Aguogui et al. [14] studied the adsorption of limonene and carvone from caraway oil onto Tunisian bentonite, showing that clay significantly delayed volatilisation and improved chemical stability. Their results highlighted MNT’s ability to be used as an effective pharmaceutical excipient for essential oil-based therapies. Similarly, Essifi et al. [15] developed montmorillonite nanoclay formulations capable of selectively retaining and releasing volatile essential oil compounds, depending on the molecular polarity and structural compatibility of the oil component with the clay matrix.

Moreover, Saucedo-Zuñiga et al. [16] investigated the use of laminar nanoclay and porous halloysite nanotubes in multilayered film composites for essential oil encapsulation. Their work confirmed the role of clays in retarding diffusion and prolonging release, thus enhancing the functional shelf life of active compounds in food and pharmaceutical applications. In a related application, Tsagkalias et al. [17] explored Na- and organo-modified MNT/essential oil nanohybrids and demonstrated their influence not only on release behaviour but also on the kinetics of in situ polymerisation, indicating the versatility of such hybrids in biomedical and materials science.

Although experimental studies have provided valuable macroscopic insights, a molecular-level understanding of the adsorption mechanisms of essential oil compounds onto clay surfaces remains limited. Computational tools, such as molecular modelling, and computational chemistry techniques, such as force fields (FFs) and density functional theory (DFT), offer a powerful approach to study clay mineral properties, intermolecular interactions, adsorption energetics, and spatial configurations at atomic resolution [18]. These techniques allow for the prediction of the vibrational binding modes, adsorption, and desorption energies of bioactive organic compounds onto clay mineral surfaces at the atomic scale, providing critical information for the rational design of clay-based drug delivery systems [19], enhancing the design of clay-based drug delivery systems [20]. These insights are particularly critical for the optimisation of formulations involving highly volatile or reactive molecules such as essential oils.

In this study, we applied a molecular modelling methodology for investigating, at the atomic scale, the crystallographic and spectroscopìc properties of the crystal structure of α-pinene and the adsorption behaviour of five anti-inflammatory terpenoids’ essential oil components—α-pinene, limonene, β-ocimene, β-caryophyllene, and β-elemene (Figure 1)—on MNT surfaces. Adsorption and desorption energies were calculated to assess the thermodynamic stability of each molecule in different adsorption states. The results of this study provide molecular-level insights into the feasibility of using MNT as a carrier system to enhance the stability, reduce the volatility, and improve the delivery potential of essential oil-based anti-inflammatory agents in skin treatments.

## 2. Materials and Methods

### 2.1. Models

The molecular structure of limonene [1-methyl-4-(1-methylethenyl)-cyclohexen] was taken from the crystal structure of a sulfonate derivative (CCDC 2312929). The molecular structures of β-elemene [(1S,2S,4R)-1-methyl-2,4-di(prop-1-en-2-yl)-1-vinylcyclohexane], β-caryophyllene [(1R,4E,9S)-4,11,11-trimethyl-8-methylidenebicyclo[7.2.0]undec-4-ene], and β-ocimene [(3E)-3,7-dimethylocta-1,3,6-triene] were extracted from PUBCHEM [21]. The molecular structure of α-pinene was extracted from the crystal structure determined by X-ray diffraction (ref. CSD-176009) [22].

The phyllosilicate employed in this study was MNT, whose crystallographic structure was adopted from a previous work [20]. Our MNT model was a dioctahedral (T-O-T) clay mineral, and its chemical composition closely resembles that of Veegum, a pharmaceutical-grade clay commonly used in pharmacological applications [23]. The clay exhibits a low degree of Al^3+^ substitution in the tetrahedral sheets and a minor presence of Mg^2+^ in the octahedral sheets, with a representative unit cell composition of Na(Al_3.17_Mg_0.83_)(Si_7.83_Al_0.17_)O_20_(OH)_4_.

For adsorption modelling, a 3 × 2 × 1 supercell was constructed with the composition Na_6_(Al_19_Mg_5_)(Si_47_Al)O_120_(OH)_24_. Each interlayer Na^+^ cation was coordinated by two water molecules. Based on prior studies of cation substitution in MNT [24], Mg^2+^ cations in the octahedral layer and Al^3+^ cations in the tetrahedral (T) layer were distributed to achieve maximal spatial dispersion. For larger adsorption systems, a 6 × 4 × 1 supercell was also used. For simulating the external surface of MNT, we included an empty space on the (001) mineral surface, applying c = 30 Å. In the hydrated models, a box with 3D periodical boundary conditions of 18 × 18 × 18 Å was filled with water molecules (200 molecules) with the Monte Carlo method using INTERFACE at a 1 g/cm^3^ density (waterbox). One molecule of the organics was placed in this waterbox for modelling the hydration states (water-org).

### 2.2. Methodology

Our calculations were conducted using empirical interatomic potentials incorporated into the INTERFACE [25] and COMPASSIII [26] FFs under three-dimensional periodic boundary conditions. INTERFACE has previously demonstrated excellent accuracy in systems similar to the one studied here [27]. Structural optimisations were carried out using the Forcite module within the Materials Studio software package [28]. The convergence was taken at a 2 × 10^−5^ kcal/mol threshold. Van der Waals interactions were evaluated using the Lennard–Jones potential, V(r) = ε[(σ/r)^12^ − 2(σ/r)^6^]. Both van der Waals and Coulombic non-bonded interactions were treated using the Ewald summation method. For the hydrated systems, three-dimensional periodic boxes containing water molecules were generated via a Monte Carlo method to achieve a density of 1 g/cm^3^. Each molecule was introduced in the centre of these waterboxes. Meanwhile, several molecular dynamics simulations were performed with INTERFACE in NVT and NPT ensembles with 1 fs steps for several simulation times: 5 ps (NVT) and 16.2 ns (NPT). In the last simulation and after the equilibration stage in the first 50,000 steps, we sampled 324 structures from 16,200,000 frames, 1 every 50,000 frames, for further analysis.

For comparison, some quantum mechanical calculations based on DFT were performed using the CASTEP code [29] with GGA (generalised gradient approximation) and PBE (Perdew–Burke–Ernzerhof) as correlation and exchange functional and norm-conserving pseudopotentials generated on the fly (OTFG) with the Koelling–Harmon relativistic treatment with periodical boundary conditions [28]. For dispersion forces, the Grimme G06 semi-empirical correction was applied [30]. This methodology was successfully used previously in similar systems [20]. After a full optimisation, a vibrational frequencies analysis was performed, assigning atomic vibration modes by means of density functional perturbational theory (DFPT) [31] by the linear response of phonon calculations with CASTEP for the Raman and infrared (IR) spectra. The IR spectra were smoothed by a Lorentzian function with a full width at half maximum parameter (FWHM) of 30 cm^−1^. The Raman spectra were simulated with a 514.5 nm incident light (Ar laser) at 298 K, applying the same Lorentzian function as above [28].

## 3. Results

### 3.1. Crystallographic and Spectroscopic Properties of α-Pinene’s Crystal Structure

**Figure 2:**
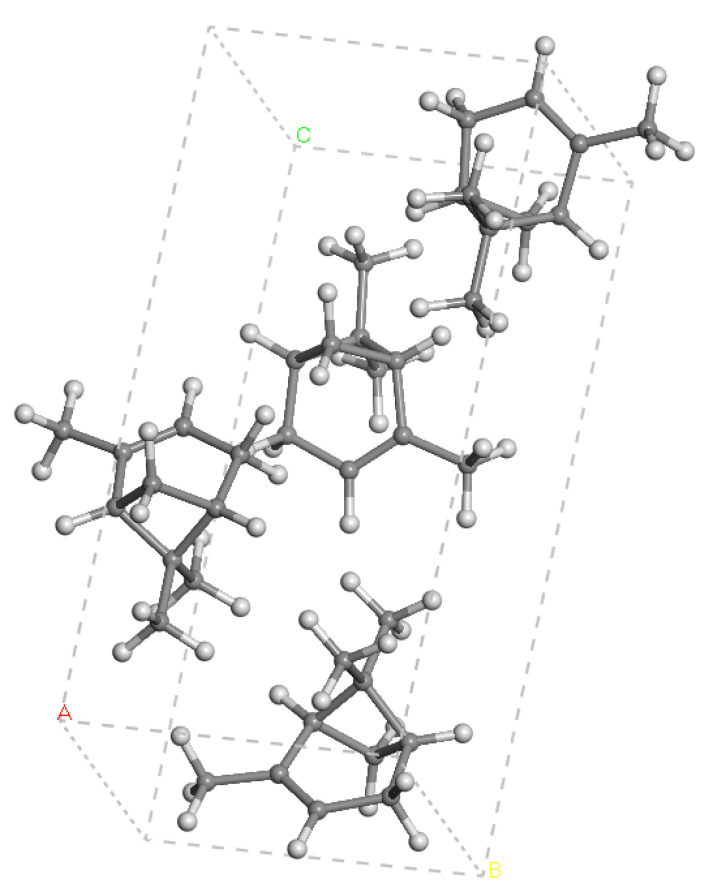
Optimised unit cell of the crystal structure of α-pinene. The H and C atoms are in white and grey colours. A, B, and C refer to the crystallographic axes.

The crystal structure of α-pinene was calculated by using both FFs, INTERFACE and COMPASS, performing full optimisation runs (atomic positions and the lattice cell parameters at a time). Both FFs yielded similar values of the cell parameters, with those of the Interface FF being slightly closer to the experimental ones. However, we found that the COMPASS FF did not reproduce nor distinguish the C=C double bond of α-pinene (d(C-C) = 1.545 Å and d(C=C) = 1.543 Å (Table 1)), whereas the INTERFACE FF distinguished both bonds quite well (d(C-C) = 1.520 Å and d(C=C) = 1.341 Å), with the experimental values being as follows: d(C-C) = 1.506 Å and d(C=C) = 1.320 Å) [22]. The computed bond lengths were slightly longer than the experimental values; however, they were below the experimental error threshold. Hence, we can consider that the experimental crystal cell parameters were reproduced quite well by the Interface FF (Figure 2). These results validate the use of INTERFACE, and it was used for the rest of this work. For comparison, we also calculated this crystal structure at the DFT level, finding similar results to those using INTERFACE (Table 1). This fact corroborates the selection of this FF for this work.

We calculated the packing energy of this crystal by comparing its energy with that of the isolated molecule. Then, the isolated molecule of α-pinene was optimised, including it in a 3D periodic box of 15 × 15 × 15 Å, which was big enough to avoid interactions with vicinal molecules. Taking into account that there were four molecules per unit cell, the packing energy was

E_pack_ = E_cryst_ − Z *×* E_mol_

where E_pack_ is the packing energy, E_cryst_ is the energy of the unit cell of the optimised crystal structure, Z is the number of molecules per unit cell, and E_mol_ is the energy of the optimised isolated molecule. Our calculations yielded a packing energy of −62.15 kcal/mol per unit cell, being −15.54 kcal/mol per molecule.

Meanwhile, the experimental spectroscopic properties were based on the vibration of the atoms of our systems, and our calculations were based on the atoms. Hence, both approaches, experimental and theoretical, were directly related. Our DFT calculations allowed us to study the spectroscopic properties of this crystal. In general, theoretical analysis of IR and Raman spectra is performed with isolated molecules and compared with experimental data [32]. However, nowadays, the experimental spectra are performed in the solid state, where the compound is in a crystal state, not as an isolated molecule. In this work, we compared the experimental data with frequency values calculated directly for the crystal structure of α-pinene for the first time. The IR and Raman spectra shown in Figure S1 (see the Supplementary Materials) were calculated for the crystal structure of α-pinene for the first time. The theoretical IR spectrum was close to the experimental spectrum of pinene ices reported in refs. [33,34]. The Raman spectrum was consistent with the experimental one for the frequency values [35].

In general, the calculated frequencies were consistent with the experimental data (Table 2). Our calculations could distinguish the vibrations of the C-H bonds of methyl and CH_2_ groups that were in the cycle of 4 C atoms from those in the cycle of 6 C atoms. The stretching ν(C-H) frequencies of those in the cycle of 4 C atoms were higher than the frequencies of those of the cycle of 6 C atoms, whereas the opposite was observed in the bending δ(C-H) frequencies. Our calculated alkenyl ν(C-H) frequencies were slightly higher than the experimental values, though both cases appeared at a higher frequency than this mode of CH_2_ or CH_3_ groups. Although a combination of several vibrations occurred in the zone of the δ(C-H) bands, we observed that the symmetric umbrella δ(C-H) mode of CH_3_ appeared at lower frequencies than the asymmetric mode.

### 3.2. Intercalation of Organic Compounds in Clay Mineral Interlayer Space

**Figure 3:**
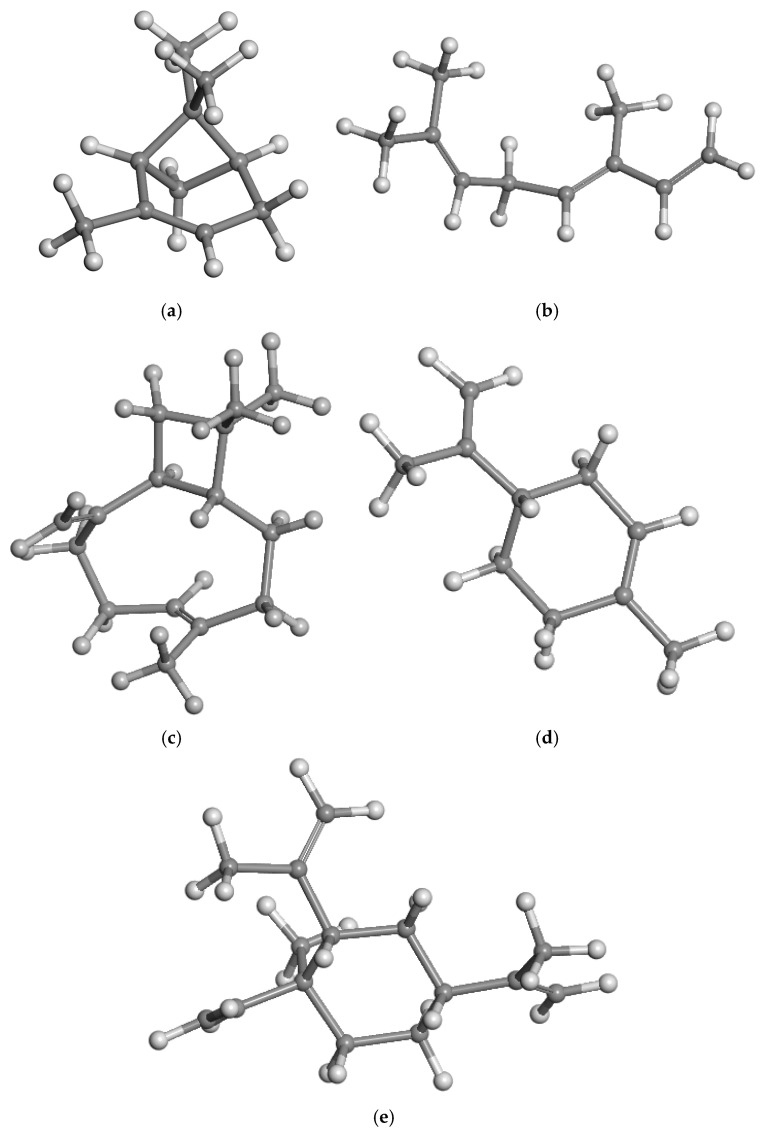
Optimised molecular structures of (**a**) α-pinene, (**b**) β-ocimene, (**c**) β-caryophyllene, (**d**) limonene, and (**e**) β-elemene.

Each isolated molecule was included in a 3D periodic box of 18 × 18 × 18 Å, being big enough to avoid interactions with vicinal molecules and optimised as an isolated molecule inside this box (Figure 3). Hence, these models represented a gas phase state of these molecules or these molecules in an inert medium. In the α-pinene molecule, the geminal methyl groups are oriented in a perpendicular plane with respect to the bicycle moiety (Figure 3a). The β-ocimene molecule maintains an extended linear orientation, where one methyl H atom is oriented towards the π-electron cloud of the terminal C=C double bond (Figure 3b). In the β-caryophyllene molecule, the terminal double bond and the methyl groups are in a perpendicular orientation with respect to the bicycle (Figure 3c). The large cycle of 9 C atoms has a C=C double bond in a trans configuration, and the vinyl and methyl substituents are in the same site of the cycle (*syn* configuration), whereas the cycle of 4 C atoms is in a trans configuration with respect to the big cycle. The isopropenyl group of limonene is close to a coplanar orientation with the ring plane (Figure 3d). In the β-elemene molecule, the cyclohexyl group is in a chair conformation, and two isopropenyl groups are in an approximately perpendicular orientation with respect to the ring plane, the same as the terminal C=C group, whereas the other isopropenyl group is close to a coplanar orientation with respect to the cyclohexenyl ring plane. This ring is in an envelope configuration (Figure 3e).

**Figure 4:**
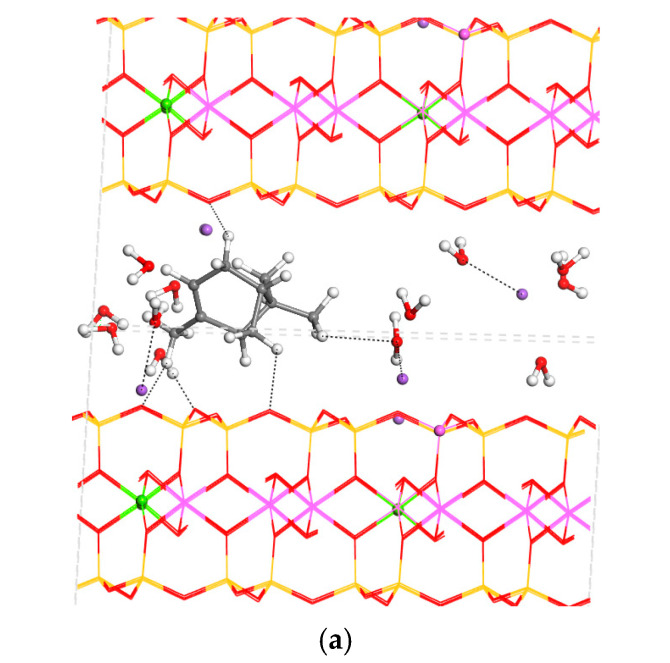

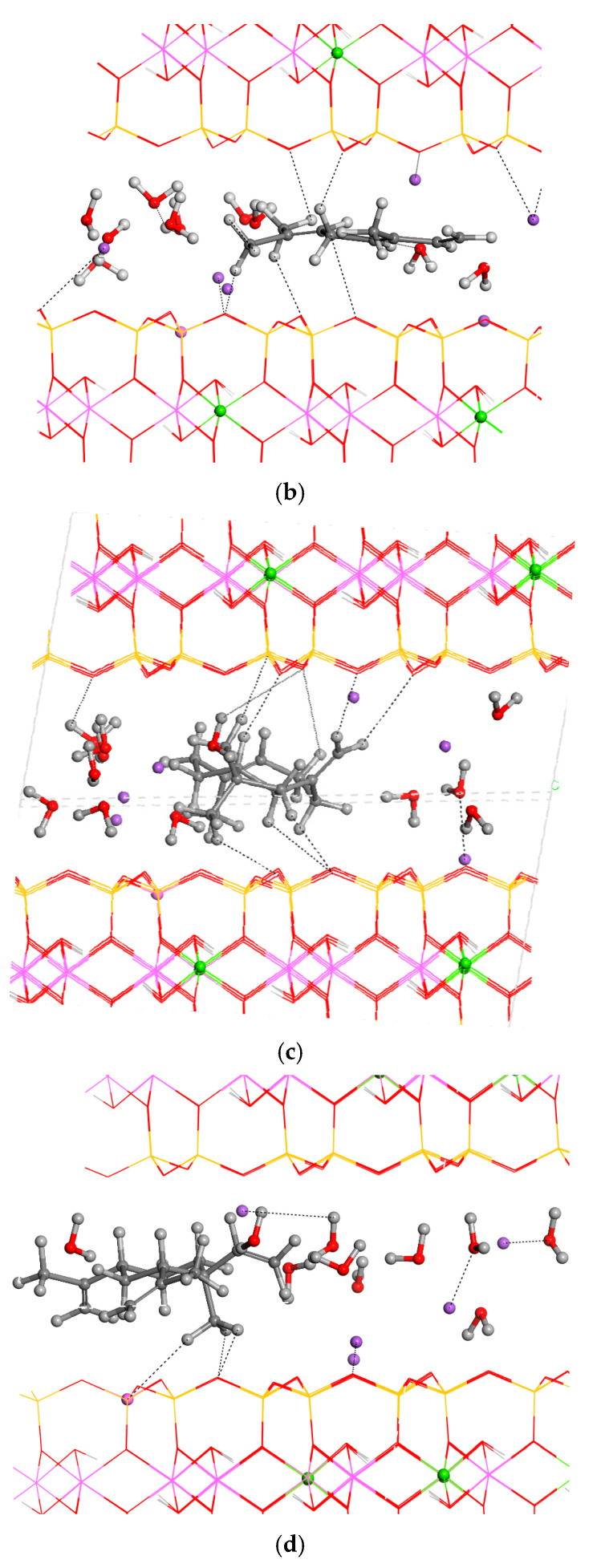

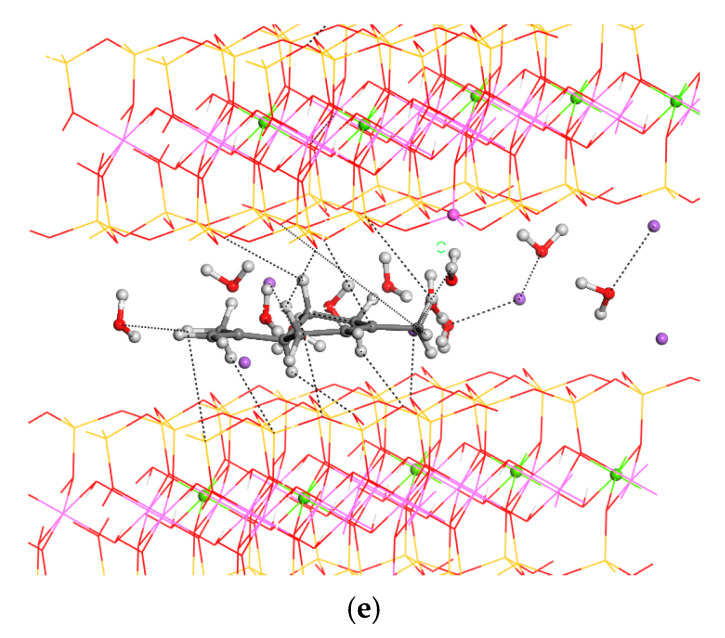
Optimised structures of MNT with intercalated organics: α-pinene (**a**), β-ocimene (**b**), β-caryophyllene (**c**), β-elemene (**d**), and limonene (**e**). Some non-bonding interactions are shown with dashed lines. The H, C, Si, Al, Mg, Na, and O atoms are in white, grey, yellow, pink, green, purple, and red colours.

The MNT structure was fully optimised, considering the atomic positions and lattice cell parameters. Each molecule was placed in the centre of the interlayer space and optimised. The crystal structure of MNT was not disturbed significantly with the interlayer adsorption of essential oil components, changing only the interlayer spacing *d*(001) that increased with the intercalation (Table 3). In all cases, some changes in the molecular structures of adsorbates were observed with respect to those of the isolated molecules. The increase in the interlayer space was greater for α-pinene and β-caryophyllene due to their bicyclic structure and higher volume. Indeed, the conformation of these bicycles and the disposition of the substituents yielded the largest cycle perpendicular to the interlayer surface opening the interlayer space. So, the α-pinene–MNT complex increased the c-axis by 2 Å with respect to MNT (Table 3 and Figure 4a). Therefore, this molecule is not placed in a parallel orientation with respect to the mineral surface, adopting a globular form, and some C-H bonds are oriented towards the mineral surface O atoms, especially those of the terminal alkenyl group. For similar reasons, the β-caryophyllene–MNT complex increased the *c*-axis by 1.6 Å with respect to MNT, ranking second in the order of our molecules in terms of increasing the c-axis (Table 3 and Figure 4c). On the contrary, the increase in the interlayer space in the adsorption of β-ocimene was very low due to the linearly extended disposition of the molecule, adopting a parallel orientation on the mineral surface, where some H atoms were oriented towards the basal O atoms of the interlayer mineral surface (d(CH…OSi) = 2.34–2.84 Å) (Figure 4b). A similar behaviour was observed in limonene, where the adsorbate adopted a parallel orientation with respect to the mineral surface, producing the smallest increase in the interlayer spacing, where the H atoms were interacting with the surface O atoms (d(CH…OSi) = 2.28–2.72 Å). Similarly, the intercalation of β-elemene into the cycle and the terminal C=C double bond were parallel to the mineral surface, where the methyl and other C-H bonds were oriented towards the mineral surface O atoms. In general, in the interlayer space, some Na^+^ cations were coordinated by water molecules and mineral surface O atoms.

**Figure 5:**
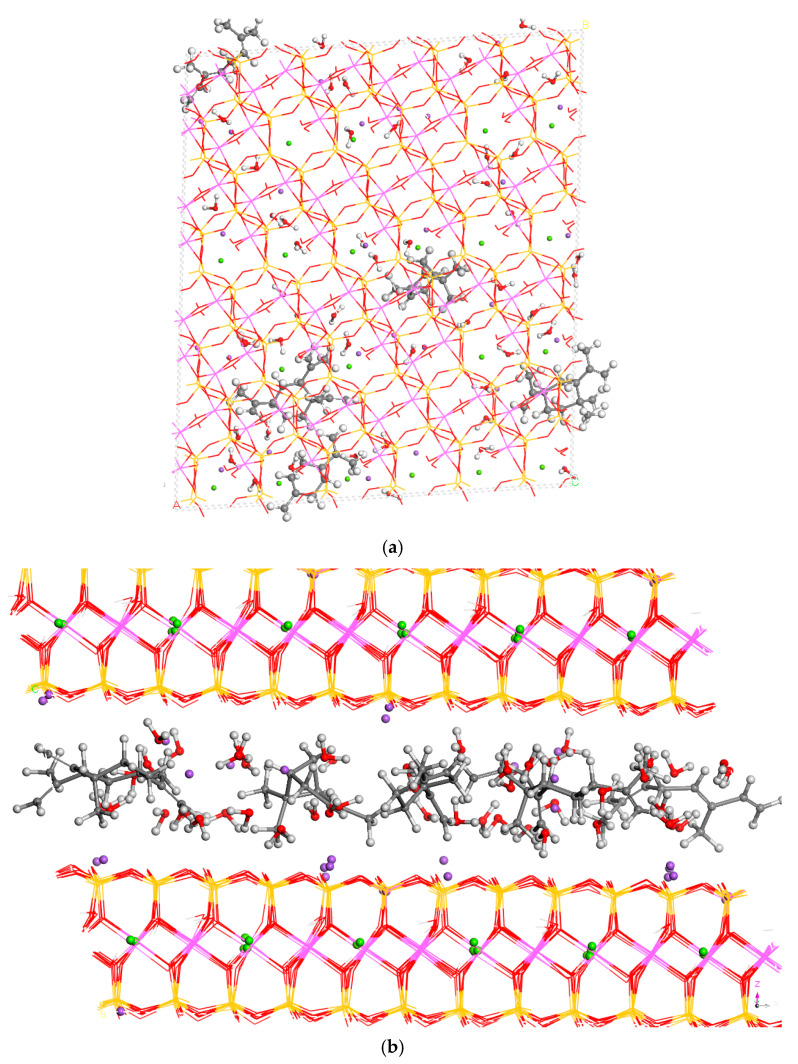
A combined hybrid composite of MNT with all adsorbates studied in this work, intercalated simultaneously, extracted from molecular dynamics simulations, and fully optimised with INTERFACE. Views in 001 (**a**) and 010 (**b**) planes.

The aims of this work were not only focused on innovative academic research, but also on more applied research, especially for possible therapeutic and clinical applications in skin treatments and thermal baths. Essential oils come from extracts of natural plants, and these extracts are complex and composed of many natural components. So, we did not use only one chemical compound in the preparation of a clay–essential oil composite, but we also introduced several compounds to the clay mineral phase. Hence, we generated a model of MNT with the simultaneous intercalation of all molecules studied in this work. For this purpose, a 6 × 4 × 1 supercell of MNT was generated with an interlayer space of c = 20 Å, and one molecule each of α-pinene, β-ocimene, β-caryophyllene, β-elemene, and limonene was intercalated randomly and simultaneously (2568 atoms) into the interlayer space of MNT (Figure 5). This combined system was fully optimised, yielding a higher interlayer space (Table 3). Between all these molecules, there were different interactions with water molecules and interlayer cations. We can see that some Na^+^ were pushed aside to the mineral surface in the tetrahedral cavities (Figure 5).

A molecular dynamics simulation was performed with this combined structure using 1 fs steps in an NVT ensemble at 298 K (5 ps; 100 samples from 5000 frames) as an equilibration phase. After this first range of dynamics for equilibrium, the two lowest-energy configurations were extracted and fully optimised. The most stable structure was used for adsorption energy calculations. This dynamics simulation continued later in an NPT ensemble at 298 K for 16.2 ns (324 structures sampled from 16,200,000 configurations) for production. In both cases, the adsorbates tended to be close together (Figure 5) due to the hydrophobicity of all these molecules. During the molecular dynamics simulations, the adsorbate molecules were close to the centre of the interlayer space (see Movie S1 in the Supplementary Materials).

From these simulations, the mean square displacement (MSD) was calculated, and the slope of the MSD with respect to the simulation time after the equilibrium time yielded the diffusion coefficient, being 0.0116 (R^2^ = 0.9982) and 0.10 (R^2^ = 0.9949) Å^2^/ps for C and O atoms, respectively (Figure S2). This indicates that the diffusion of the water molecules was much higher (by almost one order of magnitude) than the adsorbates in the confined interlayer space of MNT. We performed a similar molecular dynamics simulation of a waterbox of 200 water molecules with the mixture of the five organic molecules. We calculated the MSD, and the diffusion coefficients were 1.24 (R^2^ = 0.9893) and 4.6 (R^2^ = 0.9945) Å^2^/ps for C and O atoms, respectively. These values were drastically higher than those in the interlayer space of MNT. This confirms that the diffusion of these organic molecules was much slower in the confined interlayer space of MNT than in aqueous media. This indicates that the delivery to the external water was not drastic but was a controlled release.

The adsorption energy was calculated following the reaction:

MNT + organic = MNT-organic

where MNT–organic is the optimised structure of MNT with the organic molecule.

The adsorption energy was calculated as

E_ads_ = E_MNT-org_ − E_org_ − E_MNT_

where E_ads_ is the adsorption energy, E_MNT-org_ is the energy of the optimised structure of the complex of MNT with the organic molecule, E_org_ is the energy of the optimised molecular structure of the essential oil component, and E_MNT_ is the energy of MNT without the organic molecule.

The calculations are expressed in Table 4 for the interlayer space and external surface, respectively. Overall, the direct adsorption energies associated with the intercalation of these organic molecules into the interlayer space of MNT were comparable and positive. This indicates that the direct intercalation process was not energetically favourable, primarily due to the hydrophilic nature of MNT and the hydrophobic character of the organic molecules (Table 4).

The computed adsorption energies for interlayer incorporation were uniformly positive, ranging from 23.04 kcal/mol for β-ocimene to 53.54 kcal/mol for β-elemene. These positive values clearly indicate that direct intercalation of the organic molecules into the interlayer space was energetically unfavourable. This can be attributed to the hydrophilic nature of the MNT interlayer space, which is typically occupied by polar or charged species, such as water and cations. The intrinsic hydrophobic character of the organic molecules studied here led to unfavourable energy contributions upon confinement within the polar interlayer environment. In addition, the intercalation produced a drastic change in the conformation of some components. So, the adsorption of linear β-ocimene and the quasi-planar limonene was less unfavourable because their intercalation did not force any conformational change. This trend is consistent with previous findings [20] on hydrophobic molecule–clay interactions and suggests limited spontaneous intercalation in the absence of mediating solvents or modifications.

Meanwhile, a simultaneous intercalation of all components was energetically more favourable, with an adsorption energy of 112.7 kcal/mol, being significantly lower than the sum of the adsorption energies of all components separately (191.8 kcal/mol). Indeed, this better intercalation energy for all organics at a time should be able to be explained by a certain stabilising interaction between the organics in spite of the large distances between the different organics in the 6 × 4 × 1 cell model (Figure 5a). The presence of some components increased the hydrophobicity of the interlayer space, acting as an organo-clay for the rest of the members in the series.

### 3.3. Adsorption on External Clay Surface

**Figure 6:**
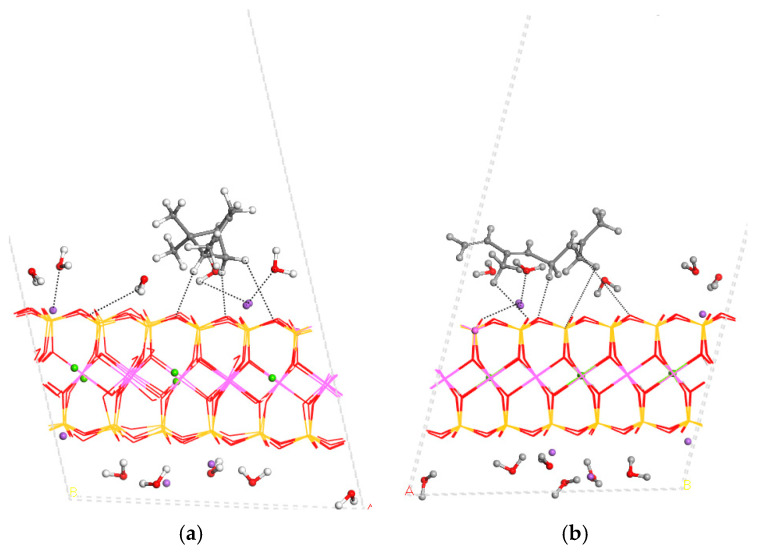

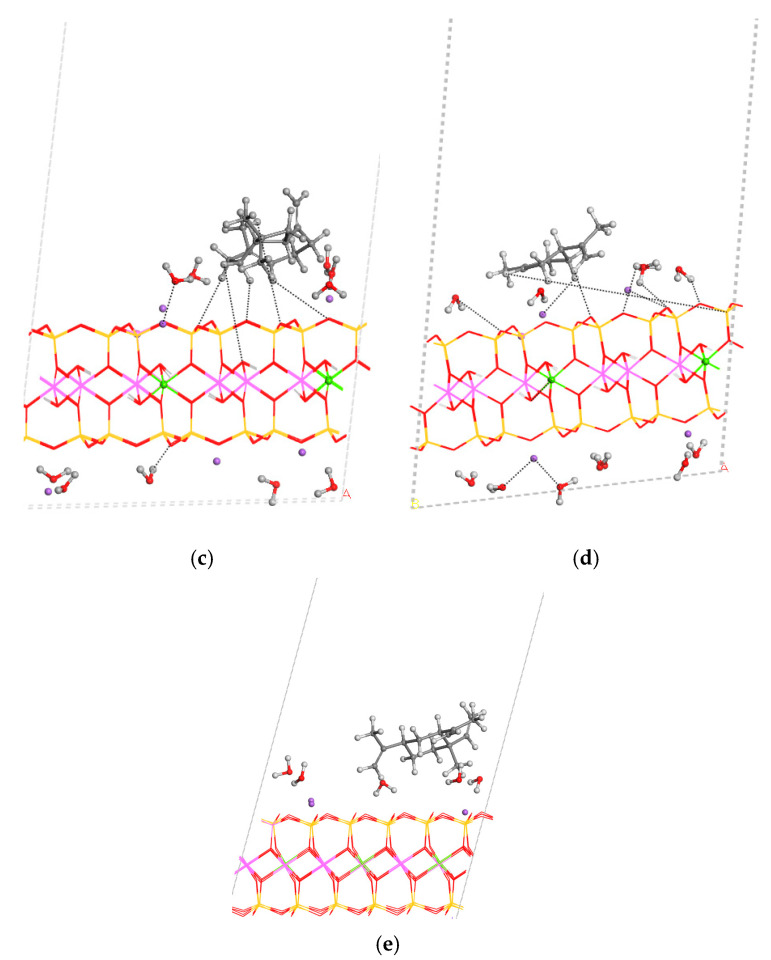
Optimised structures of the adsorption complexes of α-pinene (**a**), β-ocimene (**b**), β-caryophyllene (**c**), limonene (**d**), and β-elemene (**e**) on the external surface of MNT.

Another alternative for adsorption is on the external surface of the clay mineral. Initially, we can create an ideal clean external surface without water molecules and Na^+^ cations. However, this surface does not exist in nature, and some water molecules and Na^+^ cations can exist close to the adsorption sites of the external surface of MNT. A model representing the external surface of MNT was constructed by introducing a vacuum space of 20 Å above the (001) basal plane (*c* = 30 Å). The organic molecule was initially positioned at 2 Å above the surface, oriented parallel to the mineral surface (001) plane. Following the geometry optimisation of these adsorption complexes, maintaining the external empty space (Figure 6), the organic molecules retained a parallel orientation relative to the MNT surface. In contrast with the interlayer space, adsorption on the external surface of MNT was found to be energetically favourable for all molecules, as evidenced by the negative adsorption energies (Table 4). All adsorbates showed similar moderately negative adsorption energies, the highest being for the limonene adsorption. These values suggest that while adsorption was generally favourable, the extent of interactions varied based on the molecular structure, conformation, and surface affinity of each component.

Overall, the results reveal that external surface adsorption was energetically more favourable than intercalation for all the studied molecules. MNT provides both types of surfaces at the same time, with the interlayer space and the external surfaces existing in the interstitial borders of grains in pores. The relative accessibility to these surfaces depends on the experimental conditions. These findings highlight the importance of considering both the physicochemical properties of the adsorbate and the structural environment of the clay substrate when designing clay–organic hybrid materials.

### 3.4. Desorption Process and Delivery of Essential Oil Components to External Media

**Figure 7:**
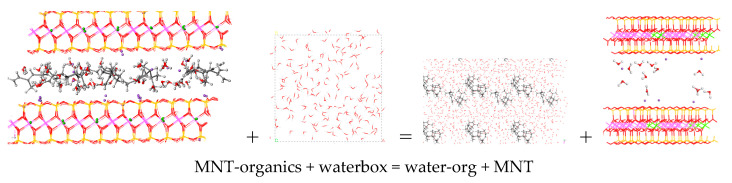
Optimised models for the desorption process. This example is for the combined case, but it is similar to all organics.

To complement the adsorption analysis, desorption energies were calculated for the same set of organic molecules from the interlayer space and the external surface of MNT clay in the presence of an explicit waterbox (18 × 18 × 18 Å, with 200 water molecules). The desorption energies provide insights into its potential use as a delivery system under aqueous conditions. The desorption process was calculated with the following equation (Figure 7):

MNT-org + water = MNT + water-org

where MNT-org is the optimised structure of MNT with the adsorbate, water is the optimised waterbox, MNT is the optimised structure of the pristine MNT, and water-org is the adsorbate molecule embedded into the waterbox system. Then, the energy was calculated with the following expression:

E_desor_ = E_MNT_ + E_wboxorg_ − E_wbox_ − E_MNT_org_

where E_desor_ is the desorption energy, E_MNT_ is the energy of the optimised pristine MNT, E_wboxorg_ is the energy of the optimised waterbox with the organic molecule included inside, E_wbox_ is the energy of the optimised waterbox system, and E_MNT_org_ is the energy of MNT with the adsorbate.

The desorption energies of organic molecules from the interlayer space and external surface of MNT are presented in Table 5. The desorption energies of organic molecules from the MNT interlayer space were consistently large and more negative for all molecule–MNT systems than those of organic molecules from the MNT external surface, indicating an energetically favourable process for the spontaneous release of these essential oil molecules to external aqueous environments like the skin and in therapeutic treatments. β-caryophyllene (−90.64 kcal/mol) and β-elemene (−90.58 kcal/mol) showed the strongest release from the interlayer space, followed by α-pinene (−63.49 kcal/mol).

The desorption energies of organic molecules from the external surface were lower, ranging from −3.47 kcal/mol (limonene) to −27.44 kcal/mol (β-elemene). All desorption energies were negative, indicating that the delivery from the external surface of MNT was also energetically favourable.

## 4. Discussion

The methodology based on the INTERFACE FF chosen in this work was validated, reproducing the crystallographic parameters of α-pinene and its spectroscopic properties. In addition, our calculations let us know the assignments of the IR and Raman spectral bands of the crystalline solid of α-pinene.

These validated methodological calculations show that the intercalation of these essential oil components into the confined interlayer space of MNT is not energetically favourable. These terpenoids are highly hydrophobic non-polar molecules with no favourable tendency to enter into the hydrophilic interlayer space of our clay mineral. This is consistent with previous experimental works on the adsorption of *Eucalyptus globulus* essential oil, containing α-pinene and β-caryophyllene, with phyllosilicates, where the adsorbed amount of these components was much lower than that of the rest of the components of the plant extract [13]. These results predict the necessity of using no solvent or non-polar solvents for the intercalation of these molecules into the interlayer space of MNT. Previous experimental works reported the adsorption of essential oils on clay minerals by using an organic solvent [13,37], without a solvent with direct vapour–solid interaction [12,15], or mixing oil–clay directly [38], aligning with our results. The diffusion of these organic components was one order of magnitude slower than water in the interlayer space of MNT, as found with our molecular dynamics simulations. This fact corroborates their difficult and slow intercalation process. Nevertheless, these calculations describe only the initial and final steps of the adsorption process without considering the temperature effect. Therefore, this adsorption will be possible under experimental conditions of time and temperature, where the Brownian movement of atoms and molecules could increase the probability of the adsorbate–mineral surface intercalation. This probability will be higher at the external surfaces in the interstitial spaces between pores, being an initial step for a possible further intercalation process. Previous experimental reports required a long reaction time, even 10 days (8.64 × 10^14^ ns). Obviously, this reaction time was outside the scope of our theoretical calculations. Our models did not include real temperature and physiological conditions or complex solvent environments typical in practical drug delivery scenarios. The study of thermodynamics, kinetic parameters, and the influence of buffers or skin mimetics at the atomic scale was outside of the scope of this work.

Meanwhile, more representative models of plant extracts, where most of the compounds studied here are present, can also be calculated. The adsorption of the components of this mixture in the same interlayer space is more probable than the adsorption of each component separately or in different interlayer spaces of MNT. The presence of some apolar molecules in the interlayer space increases the hydrophobicity of this space, facilitating the adsorption of more apolar components. This is consistent with previous experimental works, where the modification of MNT with additives forming organo-clays enhanced the adsorption capacity of these systems. However, we explored the simplest systems, without using additives and avoiding possible toxicological problems.

Nevertheless, our calculations predicted the possible increase in the *d*(001) spacing produced by the intercalation of some of these compounds. This fact can be useful information for future experimental works on powder X-ray diffraction (XRD) analysis of the adsorption of essential oil components. Furthermore, our calculations indicated that adsorption of these compounds on the external surfaces of MNT is more favourable than intercalation. The hydrophobicity of the tetrahedral silanol layers of the external surfaces of this clay mineral can be more favourable for these adsorbates. This result also predicts the possibility of adsorption of these organic compounds on the external surfaces of the interstitial pores of clay minerals without observing variation in the *d*(001) spacing by XRD experimentally. This phenomenon was observed in previous adsorption experiments on essential oils and MNT, where the adsorption was on the external surface [12].

These essential oil components are stable in the interlayer space of MNT. This means that these hybrid organic–MNT composites can be handled during their applications in skin treatments. Additionally, our calculations predicted that these bioactive compounds with therapeutic properties can be delivered to external aqueous media with an energetically favourable process. This fact can facilitate the release of these compounds to the skin surface in treatments, with possible application in warm thermal baths. Our calculations can enable experimental groups to perform release research on these systems with the kinetics of delivery and the bioactivity of post-release media.

## 5. Conclusions

The combination of the theoretical methodology used in this work allowed us to calculate the crystallographic parameters and IR and Raman spectra of the crystals of α-pinene for the first time and reproduce the known experimental data. Adsorption of some of the main components of the most-used essential oils onto the surfaces of montmorillonite was studied in this work by means of molecular-modelling methods. We selected the terpenoid components of essential oils without hydroxy groups belonging to a hydrophobic compound series. In general, the intercalation of these molecules into the confined interlayer space of montmorillonite was not energetically favourable due to the hydrophilicity of the interlayer space. However, the adsorption of these compounds on the external surfaces was more favourable, owing to the hydrophobicity of the silanol layers of the external surface of montmorillonite. We have to consider that our calculations did not include temperature and only took into account the initial and final steps of the adsorption process. Therefore, this adsorption will be possible under experimental conditions of time and temperature, which will increase the probability of the adsorbate–mineral surface interaction. Moreover, the adsorption of a real mixture of these components within an essential oil is more energetically favourable than adsorbing each pure component separately. Hence, our calculations predicted that these adsorbates can be adsorbed in the phyllosilicate clay mineral by using non-polar solvents or without solvents. The molecular dynamics simulations indicated that the diffusion of organics was one order of magnitude slower than water in the interlayer space of MNT, predicting the high stability of these guest compounds in the confined interlayer space of MNT. Meanwhile, our calculations predicted that these components can be easily delivered to the aqueous media, indicating these composites as good candidates for drug delivery bioactive compounds for therapeutic skin treatment, especially for specialised thermal baths.

## Figures and Tables

**Table 1:** Experimental [22] and calculated main geometrical features of the crystal structure of α-pinene (distances in Å). In all cases, the α and angles are 90°.

Geometric Features	*a*	*b*	*c*	d(C-C)	d(C=C)
Experimental	7.19	7.59	15.92	1.506	1.320
Calculated INTERFACE FF	7.10	7.59	15.73	1.520	1.341
Calculated COMPASS FF	7.19	7.46	15.34	1.545	1.543
Calculated DFT	7.13	7.57	15.79	1.554	1.345

**Table 2:** Values calculated by DFPT and experimental IR frequencies of the main vibration modes (in cm^−1^) of α-pinene.

Mode ^a^	Exp. ^b^	Calculated
ν(C-H) (HC=C)	3022, 3023 ^f^	3079, 3078
ν(C-H) CH_2_ ^c^	2990 ^f^	3050 _as_, 2985 _s_
ν(C-H) CH_3_	2968-2938 _as_^f^, 2874 _s_^f^	3025-2964 _as_, 2925-2922 _s_^c^, 2906 _s_^d^
ν(C-H) cyclic	2918 ^f^	2943
ν(C-H) CH_2_ ^d^	2982-2837, 2832 _s_^f^	2888 _s_
ν(C=C)	1653, 1659 ^e^, 1661 ^f^	1651
δ(C-H) CH_2_	1443, 1469-1443 ^e^ 1472-1442 _s_^f^, 1265-1204 ^e^, 1337-1205 ^f^	1470-1465 _s_^d^, 1409-1406 _s_^c^, 1190
δ(C-H) CH_3_	1371, 1333, 1380-1367 _umb_^e^, 1449 ^f^, 1381-1363 _umb_^f^	1452-1416 _as_, 1354-1349 _umb_
δ(C-H) CH	1261, 1211, 1124 ^e^, 1249-1126 ^f^	1308-1200, 1171-1029

^a^ exp. = experimental, *ν* = stretching mode, *δ* = in-plane bending mode, s = symmetric, as = antisymmetric, and umb = umbrella. ^b^ Experimental values [36]. ^c^ Cycle of 4 C atoms. ^d^ Cycle of 6 C atoms. ^e^ Experimental data [35]. ^f^ Experimental data at 50 K [33,34].

**Table 3:** Crystallographic parameters of the crystal structures of MNT with the adsorbates optimised with INTERFACE (distances are in Å, and angles are in degrees).

Cell Parameters	*a*	*b*	*c*	*α*	*β*	*γ*	*d*(001)
MNT	15.46	17.87	12.0	97.4	105.4	90.1	11.6
α-pinene–MNT	15.48	17.88	14.26	87.9	103.0	90.1	13.90
β-ocimene–MNT	15.48	17.88	13.26	106.3	103.3	90.0	12.90
β-caryophyllene–MNT	15.48	17.89	13.87	100.7	98.9	90.1	13.87
Limonene–MNT	15.48	17.89	13.19	91.1	110.7	90.1	12.34
β-elemene–MNT	15.48	17.88	13.60	93.7	100.6	90.1	13.37
Combined–MNT	31.46	36.90	15.69	108.7	103.4	90.7	15.26

**Table 4:** Adsorption energies (in kcal/mol) of the organic molecules in the interlayer space and external surface of MNT.

Adsorbate	Interlayer E_ads_	External Surface E_ads_
α-pinene	38.55	−10.14
β-ocimene	23.04	−7.42
β-caryophyllene	42.49	−10.4
Limonene	34.13	−14.72
β-elemene	53.54	−9.6
All combined	112.7	

**Table 5:** Desorption energy (Edes.) (in kcal/mol) of the organic molecules from the interlayer space and external surface of MNT.

Organic Molecule	Interlayer Edes.	Surface Edes.
α-pinene	−63.49	−14.8
β-ocimene	−55.33	−7.33
β-caryophyllene	−90.64	−21.28
Limonene	−52.32	−3.47
β-elemene	−90.58	−27.44
Combined one	−231.51	

## Data Availability

The original contributions presented in this study are included in this article. Further inquiries can be directed to the corresponding author.

## Supplementary Materials

The following supporting information can be downloaded at https://www.mdpi.com/article/10.3390/nano15201573/s1. Figure S1: Calculated IR (a) and Raman (b) spectra of the α-pinene crystal. Figure S2: Mean square displacement of the C (a) and O (b) atoms of the molecular dynamics simulation of the combined mixture of essential oil components intercalated into the MNT interlayer space (16.2 ns at NPT). The red line is the linear fitting for calculating the slope of the curve and the diffusion coefficient. Movie S1: Trajectory of the molecular dynamics simulation of the combined mixture of essential oil components intercalated into the confined interlayer space of MNT.
