# Supplementary material for: Molecular Modelling of the Adsorption and Delivery of α-Pinene and Similar Terpenes of Essential Oils on Montmorillonite Surfaces
Source: Nanomaterials (Basel). 2025 Oct 16;15(20):1573. doi: 10.3390/nano15201573 (PMC12567106; doi:10.3390/nano15201573)
Supplement: Supplementary file 1 [file nanomaterials-15-01573-s001.zip › Supplementary Figures.pdf]

Molecular Modelling of the Adsorption and Delivery of α-Pinene and Similar Terpenes of Essential Oils on Montmorillonite Surfaces

Shamsa Kanwal [1,2,3], Alfonso Hernández-Laguna [2] and C. Ignacio Sainz-Díaz [2,*]

[1] Department of Innovative Technologies in Medicine and Dentistry, University "G. d'Annunzio" of Chieti-Pescara, Via dei Vestini 31, 66100 Chieti, Italy;
shamsa.kanwal@studenti.unich.it
[2] Instituto Andaluz de Ciencias de la Tierra, IACT-CSIC, Av. De las Palmeras, 4, 18100 Armilla, Spain;
a.h.laguna@csic.es
[3] Departamento de Farmacia y Tecnología Farmacéutica. Facultad de Farmacia, Universidad de Granada, 18071 Granada, Spain
* Correspondence: ci.sainz@csic.es

SUPPORTING INFORMATION

Movie S1.- Trajectory of the molecular dynamics simulation of the combined mixture of essential oil components intercalated into the confines interlayer space of MNT (attached file).

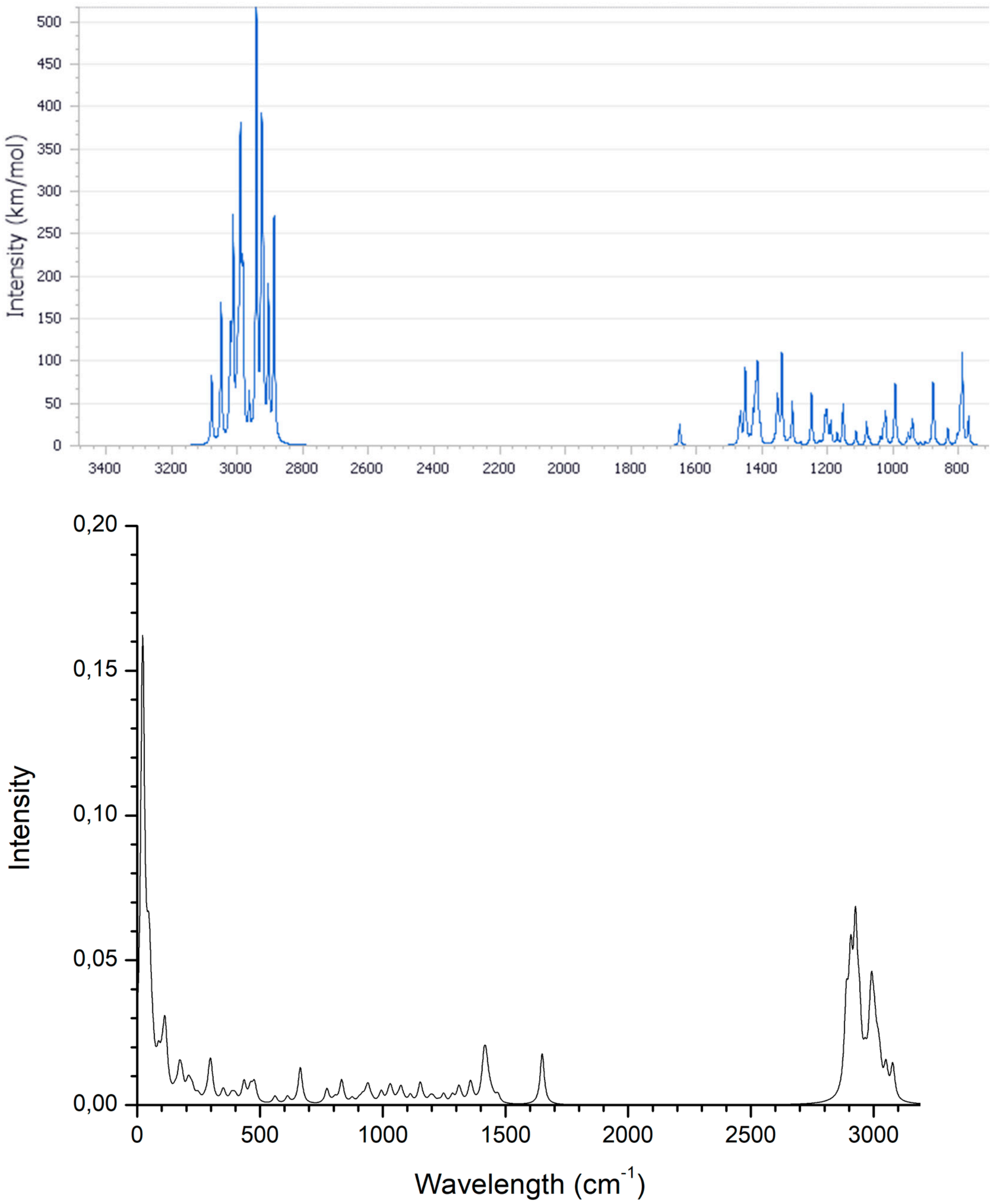

Figure S1. Calculated IR (a) and Raman (b) spectra of the α-pinene crystal.

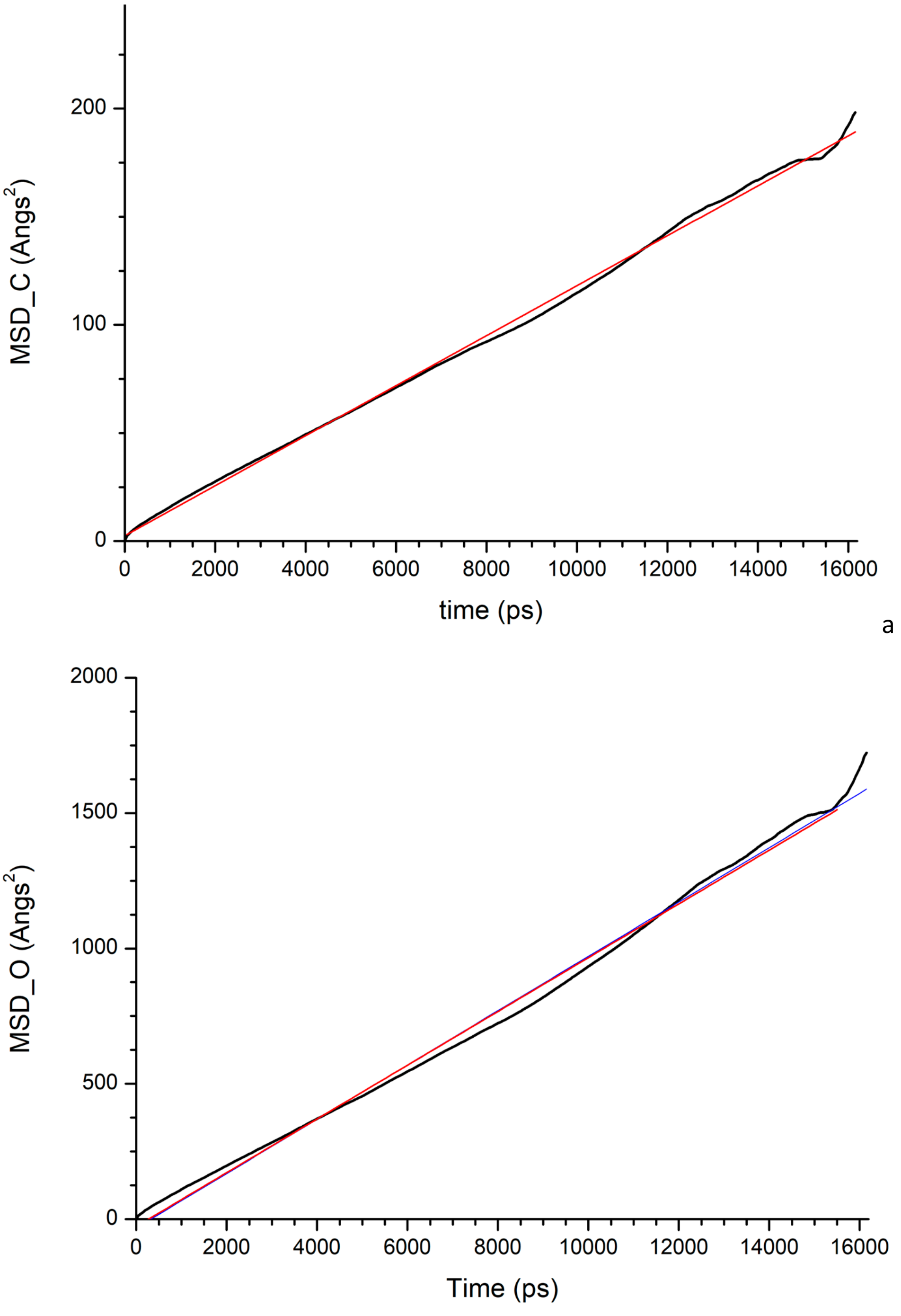

Figure S2. Mean Square Displacement of the C (a) and O (b) atoms of the molecular dynamics simulation of the combined mixture of essential oil components intercalated into MNT interlayer (16.2 ns at NPT). The red line is the linear fitting for calculating the slope of the curve and the Diffusion coefficient.
